# The mechanisms of action of metformin

**DOI:** 10.1007/s00125-017-4342-z

**Published:** 2017-08-03

**Authors:** Graham Rena, D. Grahame Hardie, Ewan R. Pearson

**Affiliations:** 1 Division of Molecular & Clinical Medicine, School of Medicine, University of Dundee, Dundee, DD1 9SY UK; 2 Division of Cell Signalling & Immunology, School of Life Sciences, University of Dundee, Dundee, DD1 5EH UK

**Keywords:** AMPK, Biguanide, Diabetes, Metformin, Review

## Abstract

Metformin is a widely-used drug that results in clear benefits in relation to glucose metabolism and diabetes-related complications. The mechanisms underlying these benefits are complex and still not fully understood. Physiologically, metformin has been shown to reduce hepatic glucose production, yet not all of its effects can be explained by this mechanism and there is increasing evidence of a key role for the gut. At the molecular level the findings vary depending on the doses of metformin used and duration of treatment, with clear differences between acute and chronic administration. Metformin has been shown to act via both AMP-activated protein kinase (AMPK)-dependent and AMPK-independent mechanisms; by inhibition of mitochondrial respiration but also perhaps by inhibition of mitochondrial glycerophosphate dehydrogenase, and a mechanism involving the lysosome. In the last 10 years, we have moved from a simple picture, that metformin improves glycaemia by acting on the liver via AMPK activation, to a much more complex picture reflecting its multiple modes of action. More work is required to truly understand how this drug works in its target population: individuals with type 2 diabetes.

**Electronic supplementary material:**

The online version of this article (doi:10.1007/s00125-017-4342-z) contains a slideset of the figures for download, which is available to authorised users.

## Introduction

**Fig. 1:**
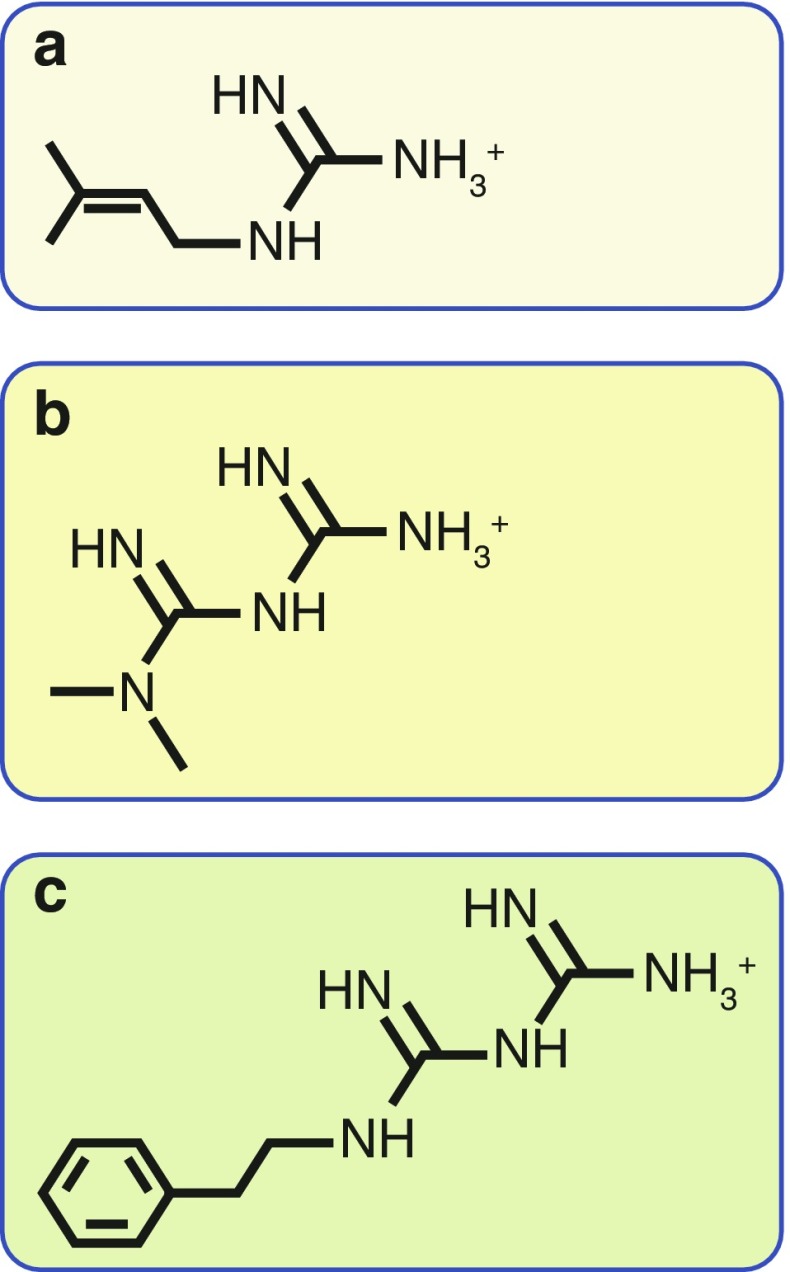
Chemical structures of galegine, metformin and phenformin. Metformin and phenformin are synthetic derivatives of galegine. Chemically, (**a**) galegine (also known as isoprenylguanidine), is an isoprenyl derivative of guanidine, while (**b**) metformin (dimethylbiguanide) and (**c**) phenformin (phenethylbiguanide) are biguanides containing two coupled molecules of guanidine with additional substitutions

Metformin and the related drug phenformin (the latter withdrawn from diabetes treatment in most countries because of side effects of lactic acidosis) are derived from galegine, a natural product from the plant *Galega officinalis*, used in herbal medicine in medieval Europe. Galegine was tested as a glucose-lowering agent in humans in the 1920s but was found to be too toxic [1, 2]. At about the same time, two synthetic derivatives of galegine, metformin and phenformin, were first synthesised and tested, although they were not introduced to clinical use until the 1950s [3]. Chemically, galegine is an isoprenyl derivative of guanidine, while metformin and phenformin are biguanides containing two coupled molecules of guanidine with additional substitutions (Fig. 1). Unlike most modern drugs, metformin is therefore derived from a natural product used in herbal medicine and was not designed to target a particular pathway or disease mechanism. It was established as a safe and effective therapy before detailed mechanistic studies became possible and, despite its clinical use for 60 years, its molecular mechanisms of action remain much debated. In this brief review, we summarise the current evidence highlighting how metformin’s benefits are likely to be caused by a variety of molecular mechanisms.

Following oral dosing of immediate-release metformin in humans, approximately 70% of the dose is absorbed from the small intestine with the remainder passing into the colon before being excreted in faeces [4]. Metformin is excreted in urine unchanged, with no metabolites reported. Plasma concentrations of metformin in humans are typically in the low micromolar range (e.g. 8–24 μmol/l) but are 30–300 times higher in jejunal samples [5]. A recent [^11^C]metformin positron emission tomography (PET) study demonstrated that oral metformin becomes highly concentrated in the intestines, liver, kidneys and bladder (reflecting its route of elimination), with only slow accumulation in muscle [6]. In this study, the hepatic tissue:systemic blood activity was ~5 following oral dosing of the metformin tracer, demonstrating that much greater metformin concentrations are achieved in the liver than in the plasma; extrapolating from systemic concentrations would estimate hepatic concentrations post-oral dose at ~50–100 μmol/l. In rats dosed with i.v. metformin, metformin accumulation was observed in the pancreas and adipose tissue at a concentration of approximately half that seen in the liver [7]; how this translates to humans is unclear. The human pharmacokinetic data point to the liver, kidney and intestines as the key target organs of metformin and in this review we will primarily focus on the liver and intestines, particularly when referring to the beneficial impact of metformin on metabolism and inflammation. Other mechanisms relating to potential cardiovascular benefits, cancer prevention and ageing are covered elsewhere in this issue of *Diabetologia* [8–10].

## Metformin and the liver

Metformin is traditionally thought to act on the liver to improve blood glucose levels and several lines of evidence support this. First, in mice lacking the organic cation transporter 1 (OCT1), which take up little or no metformin into the liver [11], metformin was ineffective at improving blood glucose after high-fat feeding [12]. Second, tracer studies in humans show that metformin lowers hepatic glucose production, with minimal impact on peripheral insulin-mediated glucose uptake. However, when only placebo-controlled studies were analysed, the impact of metformin on endogenous glucose production (EGP) was not significant unless concomitant drug-induced reductions in plasma insulin were used to ‘adjust’ EGP [13]. Third, as will be summarised here, multiple studies in mouse hepatocytes and transgenic mice provide evidence for a role of metformin in reducing hepatic gluconeogenesis and/or insulin sensitivity.

### Metformin and the mitochondrial control of hepatic gluconeogenesis

**Fig. 2:**
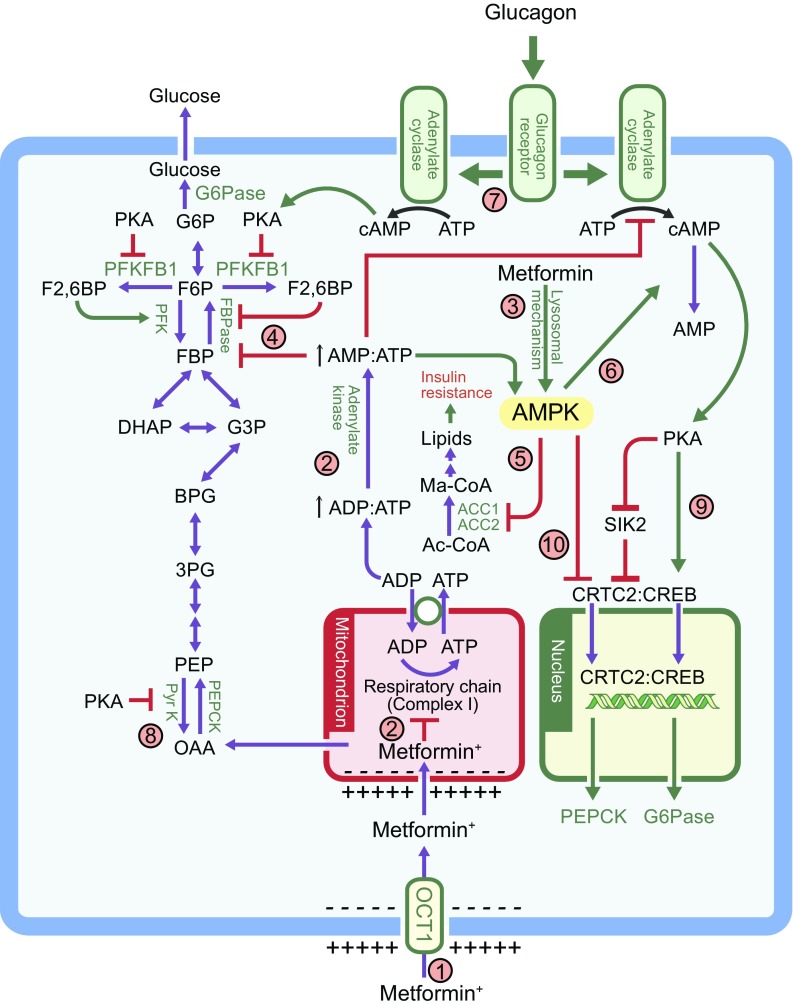
The multiple mechanism via which metformin affects liver metabolism. Note that the possible effect of metformin on mitochondrial glycerophosphate dehydrogenase [7] has not been included. (1) Uptake of metformin into hepatocytes is catalysed by the organic cation transporter-1 (OCT1) [11]. Being positively charged, the drug accumulates in cells and, further, in the mitochondria because of the membrane potentials across the plasma membrane and the mitochondrial inner membrane [14]. (2) Metformin inhibits Complex I, preventing mitochondrial ATP production and, thus, increasing cytoplasmic ADP:ATP and AMP:ATP ratios (the latter by displacement of the adenylate kinase reaction); these changes activate AMPK [17]. (3) Alternatively, AMPK may be activated by a lysosomal mechanism, not shown in detail here but requiring Axin and late endosomal/lysosomal adaptor, MAPK and mTOR activator 1 (LAMTOR1) [27]. (4) Increases in AMP:ATP ratio also inhibit fructose-1,6-bisphosphatase (FBPase), resulting in the acute inhibition of gluconeogenesis [30], while also inhibiting adenylate cyclase and lowering cAMP production [32]. (5) Activated AMPK phosphorylates the ACC1 and ACC2 isoforms of ACC, inhibiting fat synthesis and promoting fat oxidation instead, thus reducing hepatic lipid stores and enhancing hepatic insulin sensitivity [34]. (6) AMPK also phosphorylates and activates the cAMP-specific 3′,5′-cyclic phosphodiesterase 4B (PDE4B), thus lowering cAMP by another mechanism [33]. (7) Glucagon-induced increases in cAMP activate cAMP-dependent protein kinase A (PKA), causing a switch from glycolysis to gluconeogenesis via phosphorylation and inactivation of PFKFB1, causing a decrease in fructose-2,6-bisphosphate (F2,6BP), an allosteric activator of phosphofructokinase (PFK) and inhibitor of fructose-1,6-bisphosphatase (FBPase). (8) PKA also phosphorylates and inactivates the liver isoform of the glycolytic enzyme pyruvate kinase (Pyr K) and (9) phosphorylates the transcription factor cAMP response element binding protein (CREB), thus inducing transcription of the genes encoding the gluconeogenic enzymes PEPCK and G6Pase. (10) Phosphorylation of CREB-regulated transcriptional co-activator-2 (CRTC2) by AMPK, or by AMPK-related kinases such as salt-inducible kinase 2 (SIK2), causes CRTC2 to be retained in the cytoplasm, antagonising the effects of PKA on the transcription of PEPCK and G6Pase [61, 62]. PKA inhibits SIK2 by direct phosphorylation at multiple sites [62]. Ac-CoA, acetyl-CoA; BPG, 1,3-bisphosphoglycerate; DHAP, dihydroxyacetone phosphate; FBP, fructose 1,6-bisphosphate; F6P, fructose 6-phosphate; G3P, glyceraldehyde 3-phosphate; G6P, glucose 6-phosphate; Ma-CoA, malonyl-CoA; OAA, oxaloacetate; PEP, phosphoenolpyruvate; 3PG, 3-phosphoglycerate

Given that gluconeogenesis is an energy-intensive process (consuming six ATP equivalents per molecule of glucose synthesised), hepatocytes need to balance the demand for ATP with supply, with the latter primarily provided by mitochondria. Metformin accumulates within mitochondria to concentrations up to 1000-fold higher than in the extracellular medium, because metformin carries a positive charge and the membrane potentials across the plasma membrane and mitochondrial inner membrane (positive outside) drive metformin into the cell and subsequently into the mitochondria (Fig. 2) [14, 15]. The most intensively studied mitochondrial action of metformin is the inhibition of Complex I of the respiratory chain [14, 16], which suppresses ATP production. A persistent criticism of this mechanism has been the high extracellular concentrations (mmol/l) required to observe rapid effects, although lower concentrations of metformin (50–100 μmol/l) do inhibit Complex I in rat hepatoma (H4IIE) cells after several hours; this delay was ascribed to the slow uptake of metformin by mitochondria [14], which has recently been observed experimentally [15]. In addition, some studies do not detect any changes in cellular ADP:ATP ratios after metformin treatment, although they can be observed with phenformin [17]. In cells carrying out gluconeogenesis, concomitant suppression of this pathway [18] might explain modest effects on ADP:ATP ratios. Other consequences of respiratory chain inhibition besides ATP production, such as changes in the NAD^+^:NADH ratio, may also contribute to the effects of metformin on gluconeogenesis [16].

Recently, an alternative mitochondrial target of metformin has been proposed [7]. EGP (primarily by the liver) was inhibited after just 1 h of i.v. administration of metformin to rats and this was associated with an increase in the lactate:pyruvate ratio, suggesting a problem with re-oxidation of cytoplasmic NADH. The glycerophosphate shuttle is one of two systems that carry reducing equivalents from the cytoplasm into the mitochondrion for re-oxidation. In cell-free assays, metformin was found to inhibit mitochondrial glycerophosphate dehydrogenase (mGPD), a key component of this shuttle. Supporting this mechanism, i.p. administration of antisense oligonucleotides against mGPD, or a global mouse knockout, were found to lower EGP and abolish the effects of metformin on plasma glucose and EGP. However, as discussed by others, inhibition of the glycerophosphate shuttle alone may not be sufficient for a sustained impact on gluconeogenesis because the malate/aspartate shuttle will compensate unless the mitochondrial membrane potential (which is maintained by the respiratory chain) also becomes suppressed [19]. Thus, the relative contributions of inhibition of mGPD and Complex I in metformin’s glucose-lowering effects need to be established, as does the possible role of its less well understood effects on membrane properties [20] and on interactions and oxidation of amino acid-bound copper ions [21–23].

### Molecular mechanisms for metformin-associated AMPK activation

Inhibition of mitochondrial function can also explain metformin’s ability to activate the cellular energy sensor AMP-activated protein kinase (AMPK). Once activated by increases in AMP:ATP and ADP:ATP ratios (indicative of cellular energy balance being compromised), AMPK acts to restore energy homeostasis by switching on catabolic pathways generating ATP, while switching off cellular processes consuming ATP (Fig. 2) [24, 25]. Since it causes a switch from synthesis of cellular nutrient stores to their breakdown, the idea that AMPK might be involved in metformin action was attractive and, in 2001, metformin was reported to activate AMPK in rat hepatocytes and rat liver in vivo [26]. Although high concentrations (500 μmol/l) of metformin were required to observe AMPK activation after brief (1 h) treatment of cells, significant effects were observed after incubation for much longer periods with just 20 μmol/l metformin, more compatible with concentrations of the drug found in the portal vein. Supporting the idea that biguanides acted by increasing cellular AMP:ATP/ADP:ATP ratios, AMPK was not activated by either metformin or phenformin in cells expressing an AMPK mutant that is insensitive to changes in AMP or ADP [17]. However, AMPK can also be activated by glucose starvation, and by low concentrations of metformin, by a different mechanism involving the formation of a complex with the proteins Axin and late endosomal/lysosomal adaptor, MAPK and mTOR activator 1 (LAMTOR1; Fig. 2), the latter being a lysosomal protein [27]. Thus, metformin might also activate AMPK by a mechanism involving the lysosome, rather than the mitochondrion.

### AMPK-dependent and -independent effects of metformin on hepatic gluconeogenesis

The first pharmacological activator of AMPK to be developed was 5-aminoimidazole-4-carboxamide ribonucleoside (AICAR), a nucleoside that is taken up into cells and phosphorylated to the nucleotide 5-amino-4-imidazolecarboxamide riboside 5′-monophosphate (ZMP), which mimics all effects of AMP on the AMPK system [28]. The finding that AICAR downregulated expression of the gluconeogenic enzymes PEPCK and glucose-6-phosphatase (G6Pase; Fig. 2) [29] initially supported the idea that AMPK activation might be responsible for the ability of metformin to inhibit hepatic glucose production. However, an important caveat is that ZMP also modulates other AMP-sensitive enzymes such as fructose-1,6-bisphosphatase, a key enzyme of gluconeogenesis that is allosterically inhibited by both AMP and ZMP [30]. Tellingly, acute treatment with metformin or AICAR inhibited glucose production equally well in hepatocytes from control mice or mice lacking both AMPK catalytic subunits in the liver, while metformin acutely improved glucose tolerance in both mouse strains [31]. Metformin did increase cellular AMP:ATP ratios in hepatocytes [31], consistent with inhibition of the respiratory chain. It seems likely that the acute inhibition of glucose production by metformin or AICAR was due to inhibition of fructose-1,6-bisphosphatase by AMP or ZMP, respectively. However, expression of mRNAs encoding G6Pase and PEPCK was also reduced by AICAR and metformin in both control and AMPK-null hepatocytes. A potential explanation for this came with a report that adenylate cyclase, which generates cAMP in response to the starvation hormone glucagon in mouse hepatocytes is (like fructose-1,6-bisphosphatase) inhibited by AMP. Thus, AMP might have an additional AMPK-independent effect, lowering cAMP and reducing expression of gluconeogenic enzymes [32]. More recently, another group has proposed an AMPK-dependent mechanism by which metformin reduces cAMP [33]: treatment of mouse hepatocytes with a more specific AMPK activator reduced glucagon-induced cAMP levels and this was traced to the direct AMPK-mediated phosphorylation of the cAMP-specific 3′,5′-cyclic phosphodiesterase 4B (PDE4B), triggering cAMP breakdown (Fig. 2).

While controversies therefore remain, it seems certain that some of the acute effects of metformin on hepatic glucose production are AMPK-independent, with inhibition of fructose-1,6-bisphosphatase by AMP being one likely explanation. However, a major long-term, clinically relevant effect of metformin is to enhance hepatic insulin sensitivity and mouse studies suggest that this is mediated by AMPK. AMPK acutely inhibits fat synthesis and activates fat oxidation in the liver by direct phosphorylation of the two isoforms of acetyl-CoA carboxylase (ACC1/ACC2) at equivalent serine residues. Knock-in mice were generated in which both serine residues were replaced by non-phosphorylatable alanine residues (ACC1-S79A and ACC2-S212A) [34]. Consistent with the prediction that this would enhance fat synthesis and reduce fat oxidation, these mice (although not obese) had elevated diacylglycerol and triacylglycerol levels in liver and muscle. Consistent with this steatosis, the mice were hyperglycaemic, hyperinsulinaemic, glucose intolerant and insulin resistant, even on a normal chow diet. When controls were placed on a high-fat diet for 6 weeks they became just as hyperglycaemic and glucose intolerant as the knock-in mice. However, while the metabolic measures of the high-fat fed control mice substantially improved after 6 weeks of treatment with metformin, those of the knock-in mice were unaffected [34]. These intriguing results suggest that metformin enhances insulin sensitivity, at least in mice, by phosphorylation of ACC1 and ACC2 (as shown in Fig. 2). Since ACC phosphorylation is abolished by AMPK knockout [31], the long-term insulin-sensitising effects of metformin appear to be mediated entirely by AMPK.

## Metformin and the intestines

It has been known for some time that the intestines may be a target organ for metformin [5, 35], with metformin increasing anaerobic glucose metabolism in enterocytes, resulting in reduced net glucose uptake and increased lactate delivery to the liver. Several recent studies have led to a renewed interest in the gut as a major site of action of metformin and three lines of evidence highlight that the liver may not be as important for metformin action in individuals with type 2 diabetes as commonly assumed. First, the glucose-lowering effect of metformin can only partially be explained by a reduction in EGP, suggesting other glucose-lowering mechanisms for metformin [13]. Second, genetic studies in humans have established that loss-of-function variants in *SLC22A1* (the gene encoding OCT1), which reduce hepatic uptake of metformin [36], do not impact upon the efficacy of metformin to lower HbA_1c_ in individuals with type 2 diabetes [37, 38]. Third, a delayed-release metformin that is largely retained in the gut, with minimal systemic absorption, is as effective at lowering blood glucose as the standard immediate-release formulation in individuals with type 2 diabetes [39].

**Fig. 3:**
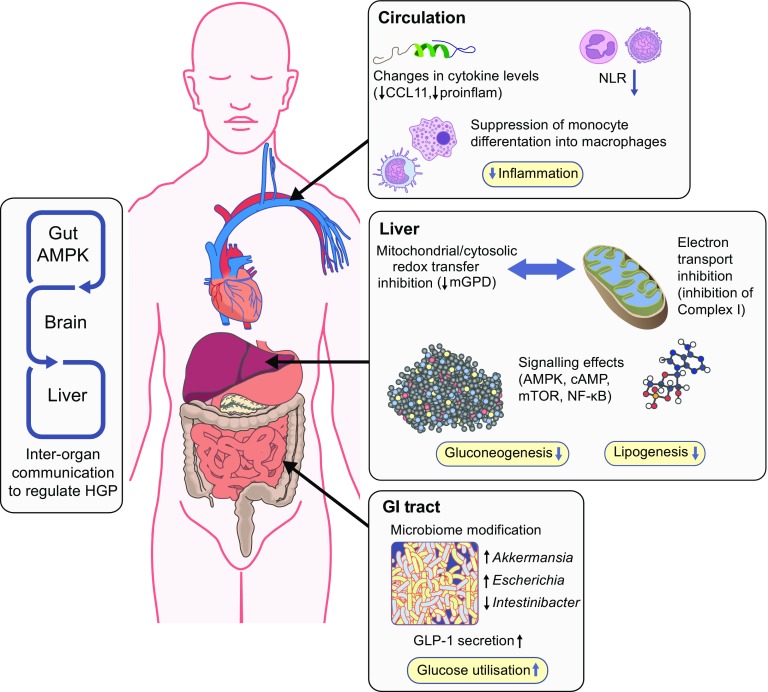
Actions of metformin on metabolism and inflammation. Responses to metformin in the blood, liver and intestines are shown schematically. In the blood, in observational studies, NLR is suppressed in humans with type 2 diabetes, whilst in randomised placebo-controlled trials, cytokines, including C-C motif chemokine 11 **(**CCL11, also known as eotaxin-1), are also shown to be suppressed with metformin treatment. Other results indicate effects of this drug on monocytes and macrophages, affecting monocyte differentiation into macrophages and proinflammatory (proinflam) cytokine secretion. In the intestines, gut metabolism, incretin (GLP-1) secretion and the microbiome are modified upon metformin use. Further, there is evidence for gut-mediated mechanism for metformin action via gut–brain–liver crosstalk, which indirectly regulates hepatic glucose output. In the liver, metformin decreases lipogenesis and gluconeogenesis, as a result of its impact on molecular signalling and on mitochondrial function. HGP; hepatic glucose production; mTOR, mammalian target of rapamycin

There are a number of putative mechanisms for how metformin could impact on glucose metabolism via actions on the intestines (reviewed in [40]). As already mentioned, metformin increases glucose utilisation by the gut; an effect that is apparent in PET imaging, where metformin-treated patients show considerable intestinal fluorodeoxyglucose (FDG) uptake, especially in the colon. A recent study in mice established that colonic FDG uptake was not increased after 48 h of metformin treatment, but was increased after 30 days of treatment, an effect that persisted despite 48 h of metformin washout [41]. The increase in FDG uptake was paralleled by an increase in AMPK phosphorylation and, like the FDG uptake, this effect was only seen in colonic enterocytes where luminal glucose was almost absent, suggesting that metformin increases colonic uptake and metabolism of systemic glucose. Metformin may also impact on glucose metabolism by increasing glucagon-like peptide-1 (GLP-1) secretion, an effect that is described for both immediate-release [42] and delayed-release [43] metformin. A further intriguing gut-mediated mechanism for metformin action was identified in rats and involves a pathway linking duodenal metformin exposure to suppression of hepatic glucose production, via the nucleus tractus solitarius and vagal efferents, through AMPK and GLP-1 receptor activation (gut–brain–liver crosstalk, Fig. 3) [44]. A final potential gut-mediated mechanism of action of metformin involves alteration of the intestinal microbiome (Fig. 3), which is outlined below in relation to inflammation; how this contributes to the glucose-lowering and gastrointestinal (GI) side effects of metformin is unknown.

### Metformin intolerance

Metformin treatment is frequently associated with GI side effects (20–30% of patients) [45] with severe side effects resulting in metformin discontinuation in ~5% of patients. The mechanism by which metformin causes GI side effects remains uncertain. However, there are a number of putative mechanisms; the side effects may simply relate to the high concentration of metformin in intestinal enterocytes, potentially explaining why slow-release formulations of metformin, which disperse slowly and reduce local luminal metformin concentrations, reduce GI intolerance. An alternative mechanism may involve serotonin, either as a result of stimulation of serotonin release from enterochromaffin cells [46], or by reducing serotonin transport via the serotonin transporter (SERT), resulting in increased luminal serotonin. Genetic studies have identified a key role for OCT1 and SERT in mediating metformin intolerance [47, 48]. A third potential mechanism of intolerance may be due to the impact of metformin on the intestinal microbiome (see later). Further studies are required to establish the mechanisms for metformin intolerance as this may enable approaches to reduce or avoid the unpleasant side effects of this drug. For example, the studies we report on the role of OCT1 in metformin intolerance would support an approach whereby OCT1-interacting drugs (such as proton pump inhibitors) are avoided in individuals experiencing GI side effects with metformin use [47].

## Inflammation, ageing and the impact of the microbiome

In the nematode worm, *Caenorhabditis elegans*, metformin lengthens lifespan through effects on intestinal microbial growth [49]. Consistent with this interesting concept of metformin’s ability to affect host metabolism indirectly, metformin expanded the gut population of *Akkermansia* spp. in animal studies, which was linked to reduced adipose tissue inflammation and suppressed postprandial hyperglycaemia [50]. More recent studies in humans found that metformin-dependent increases in *Escherichia* spp. and decreases in *Intestinibacter* spp. were the most consistently observed effects on the microbiome across datasets from different countries [51]. This recent work emphasises that microbiome changes in type 2 diabetes are predominantly associated with metformin, rather than type 2 diabetes itself, although their role as cause or consequence of therapeutic benefit requires further investigation. Metformin has, for example, been shown to have direct effects on inflammation, including effects on NF-κB signalling and differentiation of monocytes into macrophages [52, 53], as well as suppressing proinflammatory cytokines from these macrophages. Consistent with this, metformin suppresses the neutrophil to lymphocyte ratio (NLR) in type 2 diabetes (Fig. 3). NLR is a marker of inflammation that has recently been found to be a predictor of all-cause mortality and cardiac events. In addition, metformin suppresses several inflammatory cytokines in human plasma in individuals without diabetes [53]. Interestingly, one of the cytokines suppressed by metformin is C-C motif chemokine 11 (CCL11), which has previously been found to contribute to age-related cellular and tissue dysfunction. It is possible that recent observations, consistent with the ability of metformin to prolong mammalian lifespan [54, 55], may, at least in part, be due to suppression of this cytokine. Metformin may also control longevity through regulation of mammalian target of rapamycin (mTOR) signalling, which is observed in mammals and *C*. *elegans* [56, 57], with AMPK-dependent and -independent mechanisms identified [57].

## Insights from genetic studies of metformin action in humans

Recently, genome-wide association studies have been undertaken to assess genetic contributions to glycaemic responses to metformin. These offer a complementary route to mouse and cellular studies and have the advantage that they may reveal the mechanisms of action of metformin in humans with type 2 diabetes without making prior assumptions about these mechanisms. These studies are covered in more detail in the pharmacogenetics of metformin review in this issue of *Diabetologia* [58], but we briefly mention here two investigations that identified novel targets for metformin action. The first study reported on a locus on chromosome 11 involving seven genes, one of which was the ataxia telangiectasia mutated gene (*ATM*) [59]; recessive mutations in this gene cause ataxia telangiectasia, a condition associated with fatty liver, insulin resistance and diabetes. The second identified an SNP in the *SLC2A2* gene, which was associated with altered GLUT2 expression in the liver and other tissues [60]. These genes were not previously thought to be involved in the mechanisms of metformin action, and clinical and mechanistic studies are ongoing to address the role of these genes in both the liver and the gut.

## Conclusions

Metformin is a complex drug with multiple sites of action and multiple molecular mechanisms. Physiologically, metformin acts directly or indirectly on the liver to lower glucose production, and acts on the gut to increase glucose utilisation, increase GLP-1 and alter the microbiome. At the molecular level, metformin inhibits the mitochondrial respiratory chain in the liver, leading to activation of AMPK, enhancing insulin sensitivity (via effects on fat metabolism) and lowering cAMP, thus reducing the expression of gluconeogenic enzymes. Metformin also has AMPK-independent effects on the liver that may include inhibition of fructose-1,6-bisphosphatase by AMP. As cell and tissue responses are not only a product of dose, but also of treatment duration and model used, we suggest that the physiological relevance of the effects of metformin identified in cells is best validated through studies carried out in vivo, ideally in humans given metformin by the oral route. Further, pharmacogenetic studies in humans, and careful physiological validation of cell-based metformin studies, focusing on intestinal, hepatic and renal effects are warranted to enable a more robust appreciation of the key mechanisms that are active in long-term treatment with metformin in humans.

## Electronic supplementary material

ESM Downloadable slideset (PPTX 499 kb)

## Abbreviations

ACC: Acetyl-CoA carboxylase
AICAR: 5-Aminoimidazole-4-carboxamide ribonucleoside
AMPK: AMP-activated protein kinase
EGP: Endogenous glucose production
FDG: Fluorodeoxyglucose
G6Pase: Glucose-6-phosphatase
GI: Gastrointestinal
GLP-1: Glucagon-like peptide-1
mGPD: Mitochondrial glycerophosphate dehydrogenase
NLR: Neutrophil to lymphocyte ratio
OCT1: Organic cation transporter 1
PET: Positron emission tomography
PKA: Protein kinase A
SERT: Serotonin transporter
ZMP: 5-Amino-4-imidazolecarboxamide riboside 5′-monophosphate
