# Supplementary material for: The mechanisms of action of metformin
Source: Diabetologia. 2017 Aug 3;60(9):1577–85. doi: 10.1007/s00125-017-4342-z (PMC5552828; doi:10.1007/s00125-017-4342-z)
Supplement: Supplementary file 1 — (PPTX 499 kb) [file 125_2017_4342_MOESM1_ESM.pptx]

## Slide 1
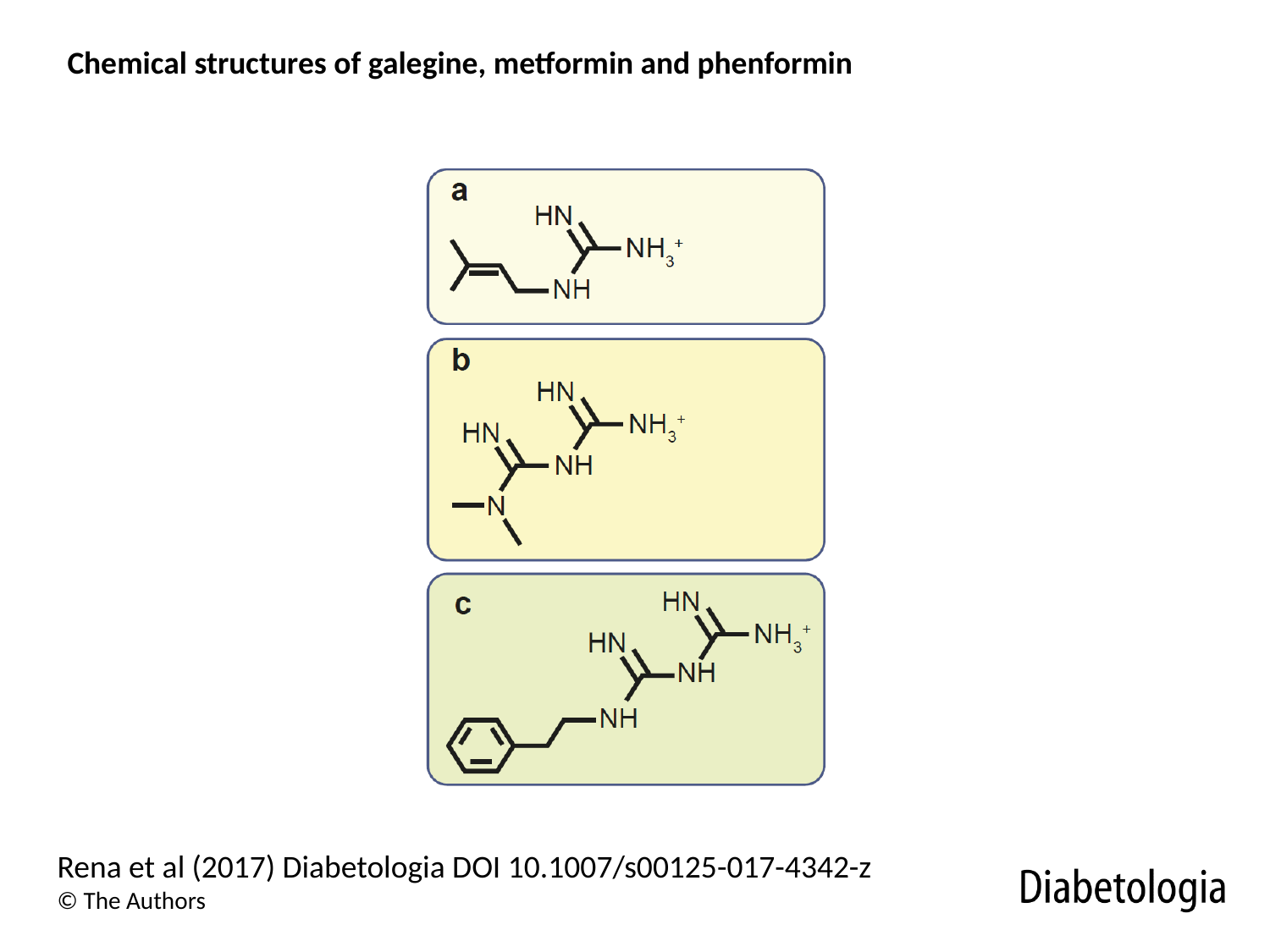

Chemical structures of galegine, metformin and phenformin
Rena et al (2017) Diabetologia DOI 10.1007/s00125-017-4342-z
© The Authors

## Slide 2
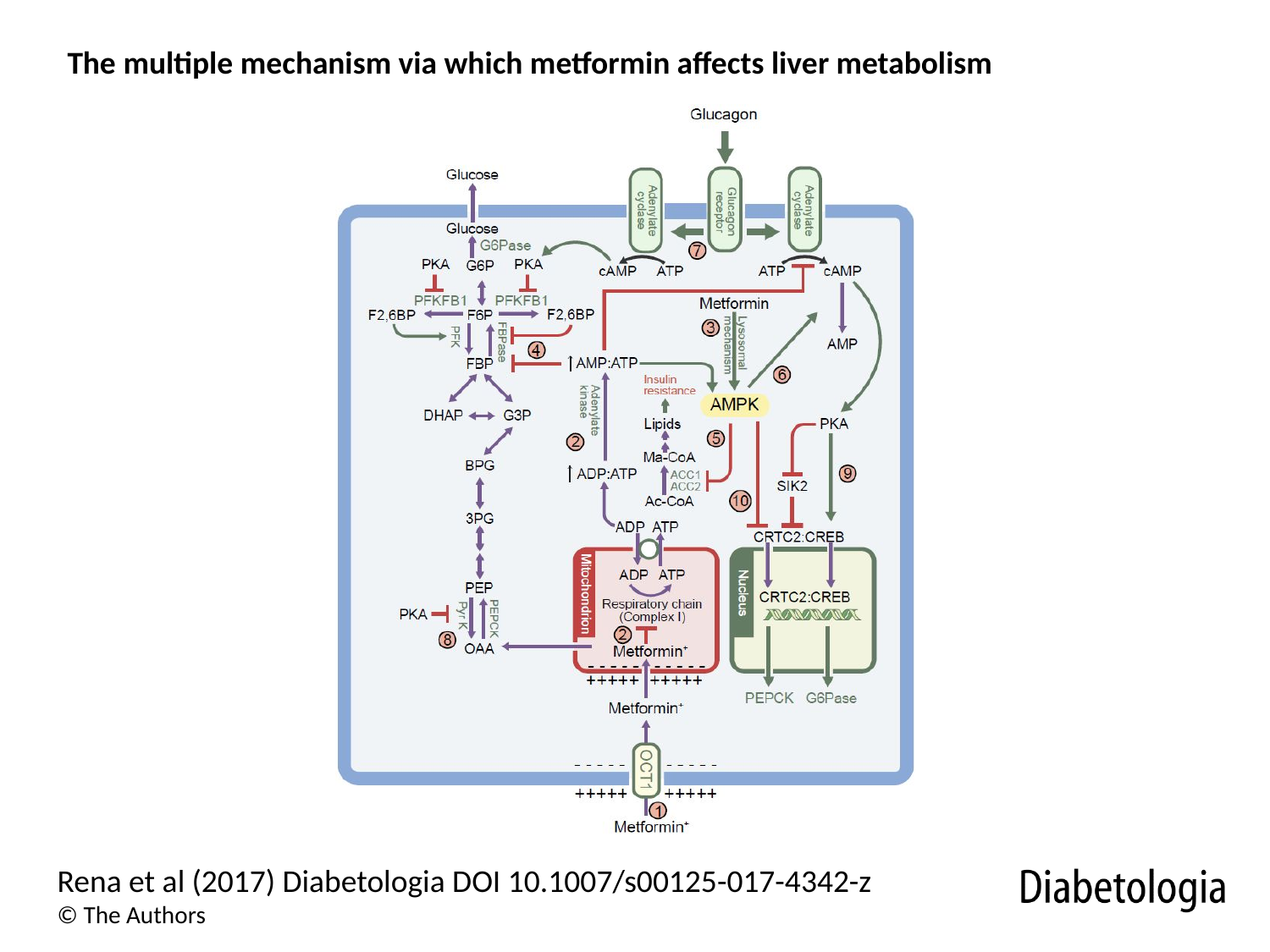

The multiple mechanism via which metformin affects liver metabolism
Rena et al (2017) Diabetologia DOI 10.1007/s00125-017-4342-z
© The Authors

## Slide 3
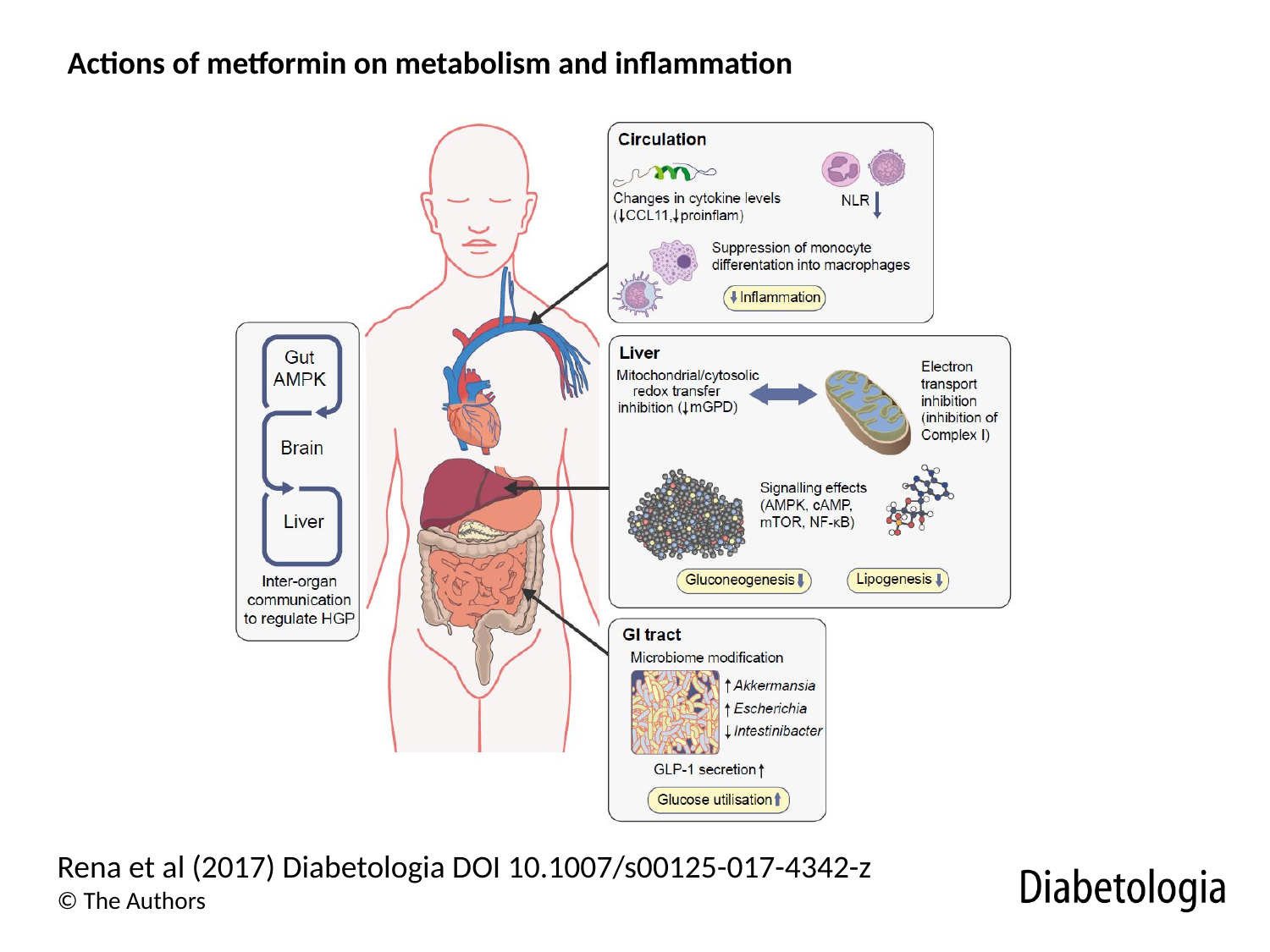

Actions of metformin on metabolism and inflammation
Rena et al (2017) Diabetologia DOI 10.1007/s00125-017-4342-z
© The Authors
